# Radiotherapy treatment scheduling: Implementing operations research into clinical practice

**DOI:** 10.1371/journal.pone.0247428

**Published:** 2021-02-19

**Authors:** Bruno Vieira, Derya Demirtas, Jeroen B. van de Kamer, Erwin W. Hans, Willem Jongste, Wim van Harten

**Affiliations:** 1 Department of Radiation Oncology, Netherlands Cancer Institute—Antoni van Leeuwenhoek, Amsterdam, The Netherlands; 2 Center for Healthcare Operations Improvement and Research (CHOIR), University of Twente, Enschede, The Netherlands; 3 Faculty of Behavioural Management and Social Sciences, Department Industrial Engineering and Business Information Systems, University of Twente, Enschede, The Netherlands; 4 Bernard Verbeeten Instituut, Tilburg, The Netherlands; 5 Department of Health Technology and Services Research, School of Governance and Management, University of Twente, Enschede, The Netherlands; MD Anderson Cancer Center, UNITED STATES

## Abstract

**Background:**

Every week, radiotherapy centers face the complex task of scheduling hundreds of treatment sessions amongst the available linear accelerators. With the increase in cancer patient numbers, manually creating a feasible and efficient schedule has shown to be a difficult, time-consuming task. Although operations research models have been increasingly reported upon to optimize patient care logistics, there is almost no scientific evidence of implementation in practice.

**Methods:**

A mathematical operations research model was adapted to generate radiotherapy treatment schedules in two Dutch centers. The model was iteratively adjusted to fulfill the technical and medical constraints of each center until a valid model was attained. Patient data was collected for the planning horizon of one week, and the feasibility of the obtained schedules was verified by the staff of each center. The resulting optimized solutions are compared with the ones manually developed in practice.

**Results:**

The weekly schedule was improved in both centers by decreasing the average standard deviation between sessions’ starting times from 103.0 to 50.4 minutes (51%) in one center, and the number of gaps in the schedule from 18 to 5 (72%) in the other. The number of patients requiring linac switching between sessions has also decreased from 71 to 0 patients in one center, and from 43 to 2 in the other. The automated process required 5 minutes and 1.5 hours of computation time to find an optimal weekly patient schedule, respectively, as opposed to approximately 1.5 days when performed manually for both centers.

**Conclusions:**

The practical application of a theoretical operations research model for radiotherapy treatment scheduling has provided radiotherapy planners a feasible, high-quality schedule in an automated way. Iterative model adaptations performed in small steps, early engagement of stakeholders, and constant communication proved to facilitate the implementation of operations research models into clinical practice.

## Introduction

Radiotherapy (RT), as a treatment modality for cancer care, has been experiencing a rising global demand [1]. In Europe, demand for radiotherapy services has been estimated to increase by 16%, in total, during the period from 2012 to 2025 [2]. External beam RT treatments are delivered by a set of linear accelerators (linacs) in a series of (daily) radiation sessions of 10–30 minutes each. With the growing demand for RT services, the number of treatment sessions to be booked amongst the available machines has been continuously increasing. This makes the problem of scheduling RT sessions increasingly complex for RT centers, which aim at managing their resources in the most efficient manner in order to provide the patient-centered care while keeping waiting times low. Therefore, several indicators, possibly distinct between RT centers, must be considered when designing a methodology for scheduling RT treatments, with timeliness, patient-centeredness, and staff satisfaction being amongst the most important ones [3]. Timeliness is crucial as long waiting times (time from referral to start of treatment) have been linked with, amongst others, higher risk of local recurrence [4], and prolonged psychological distress in cancer patients [5]. Patient-centeredness relates to not only providing the best care for each specific patient based on their personal and medical needs, but also relates to maximizing patient satisfaction levels regarding their treatment options. Results of a survey amongst six RT centers showed that patients have different preferences regarding the time of their treatment appointments, with 80% of the patients agreeing that having RT between 08h00 and 16h30 was preferred, while 14%-15% preferred the 07h30-08h00 or the 16h30-17h00 time windows. A further 6% or fewer patients preferred times before 07h30 or after 18h00 [6]. From a patient’s perspective, research shows that the professional staffing standards and low waiting times for both diagnosis and treatment are the most important factors [7]. To keep quality of labor high, a predictable work schedule and an appropriate amount of assigned workload are necessary from a staff viewpoint. Every week, RT centers are faced with the problem of scheduling hundreds of treatment sessions on the available linacs [8]. Given the high number of technical and medical constraints to be considered for each patient (e.g. start treatment within the due date, patient allocation to specific machines, etc.), the manual execution of such a schedule is a difficult, time-consuming task that often leads to solutions that are far from optimal. Therefore, the development, validation, and implementation of scheduling algorithms can be a solution to support RT centers to schedule radiation sessions in an optimized manner regarding the relevant performance indicators.

Operations research (OR) is a discipline that includes a range of techniques aimed at improving decision-making processes in many areas. Combining knowledge from applied mathematics, computer science, and industrial engineering, OR methods such as computer simulation [9] and mathematical programming [10] have been widely used to propose solutions for complex real-world problems, including the health care field [11,12]. Recent studies have shown that there is very little scientific reporting on implementation of OR-based algorithms in clinical practice [13,14]. Moreover, several OR-based models have been developed to solve RT treatment scheduling problems, as shown by a literature review by Vieira *et al*. [15]. However, their review also revealed that none of the 18 reviewed papers reported a (pre-)implementation of the model, suggesting that implementation rates of OR approaches in RT are rather low. The inherent complex nature of the optimization problems, the impact on organizational changes, the involvement of several specialized personnel (such as operations research specialists, IT, managers and clinicians) and the need for developing a stable, user-friendly and updated decision support system contribute to the challenging nature of the implementation process. A scoping review found that poor availability of representative data of sufficient quality, and a lack of collaboration between those who develop OR models and relevant internal stakeholders were found to be common challenges for effective OR modelling in global health [16].

In this paper, we study the implementation of a weekly schedule for radiotherapy treatments obtained by using an earlier developed mathematical programming model [17]. In our previous publication, we proposed a theoretical model and tested it using fictitious data generated from datasets of a one-year period. In the current paper, we not only use real data from an actual planning week instead of fictitious data to test our model, but we also have the direct collaboration of two real-world RT centers in all major steps such as data gathering, model adjustment, and validation of the obtained solutions. According to our findings, this is the first study to involve the participation of managers and clinicians from different real-world radiotherapy centers (Vieira et al. 2016). The model is iteratively adjusted together with planners and clinicians to meet the constraints and objectives of each RT center, with the resulting computer program being able to find a good quality schedule in reasonable computation time. We compare the performance of the final schedule output by the model with the solution that was manually constructed in practice and elaborate on the main limitations found during the model adjustment process. By having the resulting schedules validated by the actual decision-makers in the clinics, we show that it is feasible to actually bring OR models towards implementation in clinical practice.

## Materials and methods

**Fig 1:**
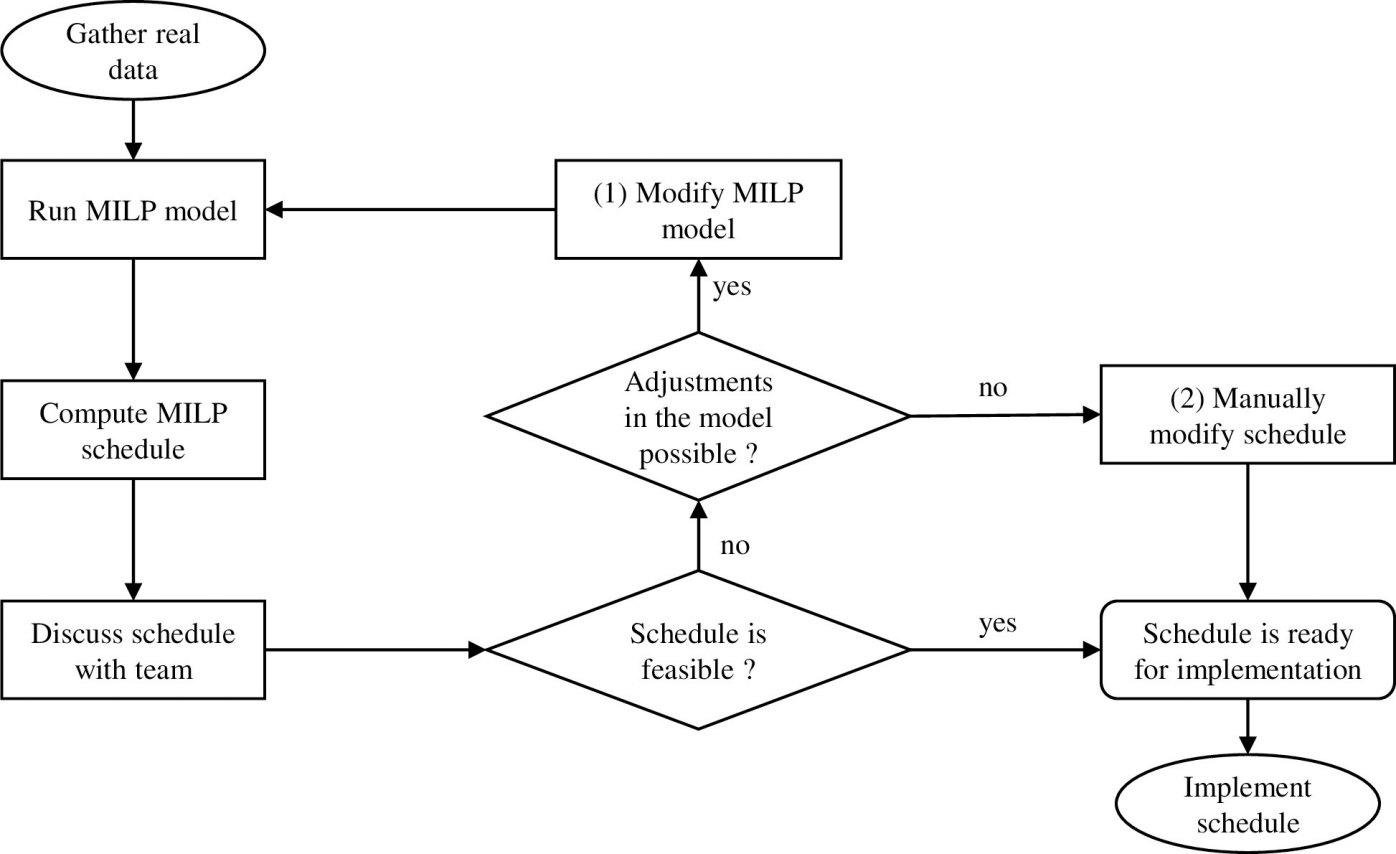
Schematic overview of the MILP-based schedule construction and validation phases before implementation.

In this study, we have used and adapted the mixed-integer linear programming (MILP) model proposed by Vieira *et al* [17] to improve the RT treatment scheduling based on historic off-line data of two Dutch RT centers. MILP is a mathematical programming framework that involves the use of integer and continuous variables to model a decision problem by means of linear inequalities [17]. The process of adjusting the existent model to ensure that it produces a feasible schedule for each RT center was completed iteratively following a structured sequence of steps (Fig 1). We started by gathering the necessary patient data for running the MILP model, and discussion meetings were performed to check on the feasibility of each obtained schedule. If modifications were necessary due to infeasibilities found in the output schedule as a result from model deficiencies, the MILP model was corrected. When changes on the MILP model were not possible, the necessary changes were then completed manually by the OR specialist and the planner in order to ensure that all constraints are met for all patients, further guaranteeing the feasibility of the planned schedule.

### The radiotherapy centers

#### Netherlands Cancer Institute–Antoni van Leeuwenhoek (NKI)

The NKI is a comprehensive cancer center that combines research and care, located in Amsterdam, the Netherlands. The RT department of the NKI treats approximately 5000 patients per year and is equipped with eight linacs divided over two locations, Amsterdam (six) and Hoofddorp (two). The department focuses its operations on patient-centered and personalized quality treatments, highly adapted to the individual. Therefore, the number of possible patient care pathways, as well as the number of different healthcare professionals (e.g. specialized RT technicians, dietitians, dentists, etc.) involved in the process is rather high when compared to an average RT center. By combining research and care, the RT department of the NKI aspires to be a frontrunner in the development and adoption of innovative (information) technologies.

#### Bernard Verbeeten Instituut (BVI)

The *Bernard Verbeeten Instituut* (BVI) is an independent RT group delivering RT treatments to patients living in the South of the Netherlands. The BVI provides around 4700 treatments per year. The main location, in Tilburg, is equipped with four linacs, while the satellite locations in Den Bosch and Breda have two linacs each. BVI is highly focused on patient care logistics and efficiency, with the goal of delivering timely, good quality treatments in a patient-friendly manner. Being early adaptors of emerging technologies, BVI has been active in fostering and implementing interventions for logistics improvement (e.g. Lean methodologies) in their clinic over the last decade. We take the Breda location as the subject of our study.

#### Information gathering

We gathered information regarding the problem specificities, boundary conditions, and objectives of each participating RT center via a series of meetings held with personnel from each institute. The NKI, being the institution that hosts the project, has allowed a higher number of professionals to be involved in the process. A group consisting of an OR specialist, a radiation oncologist, an appointment planner, the head of the appointment office, and a medical physicist were involved in the project, in which only the Amsterdam location (six linacs) was considered. In the BVI, a logistics specialist and the head of medical physicists have been working together with an OR specialist to adjust and validate the model for the Breda location (two linacs). A series of five face to face meetings with members from both centers, dozens of “on-site” interviews with managers and clinicians, and hundreds of e-mail exchanges clarifying details over the course of two years, facilitated the necessary steps (problem definition, data gathering, model development/adjustment, and schedule validation) for the application of the OR model.

### Mathematical model description

The MILP model outputs a schedule for treatment sessions of a pre-defined number of patients amongst a set of linear accelerators with a certain daily capacity, divided in time slots of five minutes. The model decides upon the specific time slot(s) of each linac to allocate to each patient on each day of a planning horizon of one week (five working days). If a certain starting slot is assigned to a patient, we prevent the next slots needed to achieve the corresponding patient’s session duration on that same linac and day from being assigned to other patients. Moreover, we use a set of binary variables to decide upon the specific time slots chosen for each patient’s session, and a set of real variables to represent the deviations from the intended sessions’ starting time for each patient, in each day. The full model, together with the description of the specific constraints, decision variables, and input parameters can be found in S1 Appendix. The model has been adapted to represent the problem specificities of each RT center by formulating an objective function according to the main objective of the center, and by removing or modifying constraints. In the NKI case, patients are given the possibility of choosing a preferential time window for the time of their treatment sessions. The objective of the NKI is then to find a schedule that maximizes the fulfillment of those patient preferences requests [17]. Thus, we use objective function (1a) of the MILP model (S1 Appendix) to maximize the number of sessions that are scheduled within the time window given by patients as a preference. All constraints (2–19) are applicable to the NKI case. In the BVI, however, patient preferences are not explicitly asked to patients to be considered in the scheduling process. In their case, the main objective is to find a schedule that minimizes the number of gaps (15 minutes or longer) in the schedule in order to minimize staff idle times (1b). Constraints (5–7) and (17–18) and variables $\Delta_{it}^{-},\Delta_{it}^{+}$ are discarded when running the MILP for the BVI case.

### Input data and planning horizon

Anonymized historical patient data has been extracted from the patient information systems of each center: MOSAIQ [18] in the NKI, and ARIA [19] in the BVI case. Table 1 presents the summary of the input data used to feed the MILP model for the targeted planning week in which the development and validation steps were performed. Patient data extracted from the databases correspond to the necessary input parameters needed to run the model, as presented in S1 Appendix. All external-beam RT patients (regular and urgent) known by the Friday prior to the planning week were included in the patient group to be scheduled. The due date of each patient is set according to the national timeliness standards [20]. Linacs down times for maintenance were also considered for both centers, with an average of 2.5 hours for scheduled maintenance per linac, per week. In the NKI, distinct linac specifications required by certain patient groups exist. Therefore, a pre-allocation of patient groups to certain linacs was considered for their case (e.g. bladder patients can only be assigned to linacs number 2 and number 6). Since patient preferences regarding appointment times were not recorded in actual practice in the NKI, their values are unknown. Therefore, we have interviewed actual planners to estimate patient preferences according to their empirical knowledge, resulting in the proportion and time windows shown in Table 1. Since maximizing the satisfaction of patient preference requests is not an objective for the BVI, the corresponding data has not been collected or estimated. This study conducted the necessary steps to comply with the applicable legislation according to the General Data Protection Regulation (GDPR). By rendering data anonymously from the patient information systems (MOSAIQ and ARIA), not traceable to any particular individual, the requirements regarding the use of data have been fully complied with. Given that no information about patients has been provided, no formal ethics approval was required to access the raw historical data. Clinicians (RTTs, radiation oncologists, managers, appointment schedulers) who participated in this study gave their informed verbal consent to participate, adhering to the applicable legislation, thus a written consent has been deemed unnecessary.

**Table 1:** Data used as model inputs for the computational experiments of each center.

	NKI	BVI
Week (planning horizon)	24–28 June 2019	9–13 December 2019
Number of patients to be scheduled	213	51
Number of patients starting treatment	48	6
Linacs	6	2
No. sessions scheduled	807	232
Linac opening times (daily capacity per linac)	07h30 – 17h00 (570 min)	08h00 – 17h30 (570 min)
Average session duration, in minutes (SD)	14.7 (3.8)	13.3 (3.1)
Patient preference distribution	07h30-09h30 (10%)	n.a.
	09h30-15h30 (60%)	
	15h30-17h30 (30%)	

### Performance metrics

Key Performance Indicators (KPIs) have been assessed through interviews conducted with the actual planners and managers of each center. In both cases, the KPIs found to be monitored during the schedule construction method are related to patient-centeredness, timeliness, and staff satisfaction (Table 2). The identified KPIs were modeled either in the form of the objective function being optimized (e.g. minimize gaps in the schedule), or in the form of constraints (e.g. ensure that all patients start treatment within the target date according to the national timeliness standards).

**Table 2:** Key performance indicators defined by the RT centers.

KPI	Description	RT center (s)
Number of gaps in the schedule	Minimize the number of empty time periods between appointments to avoid staff idle times (only time intervals of 15 minutes or longer are considered)	BVI
Number of sessions within the desired window	Maximize the fulfillment of patient requests regarding the time window indicated as the most preferred: 07h90-09h30, 09h30-15h30, or 15h30-17h30	NKI
Consistency of appointments	Minimize the (average) deviation between sessions, in order to allow the patient set up a routine for their sessions	BVI, NKI
Number of patients switching linacs during treatment	Minimize the number of patients switching between different linacs in order to have the patient receive treatment with the same personnel and facilities	BVI, NKI
Fulfillment of the maximum waiting time targets defined by the Dutch Society for Radiation Oncology [20]	Ensure that patients fulfill the national standards: acute patients should be treated within one day, subacute patients should start treatment within 10 days, and regular patients should start treatment within 28 days.	BVI, NKI

### Model validation

The schedules generated by the various versions of the MILP model have been validated by the planners of the corresponding center by cross-checking actual patient and resource information data to confirm that the final schedule is ready for implementation. Since the task of assessing all technical and medical constraints for all patients showed to be too complex and time-consuming, we have selected a random sample of 20 patients in the NKI, and 10 patients in the BVI, from several tumor sites, to perform the validation check. We continued to perform model iterations until all inconsistencies with daily practice were tackled and no further improvements were considered likely by the responding teams. This resulted in the final MILP model.

For the final test, the treatment requirements (e.g. number of sessions, frequency, due date, etc.) associated with each of the corresponding sample of patients have been validated by matching them with actual values. Moreover, the weekly schedule of each linac produced by the final model has been analyzed by the managers and planners of each center in order to look for possible infeasibilities regarding the distribution of appointment sessions, idle times, linacs’ down times for maintenance, or the management of staff. The schedules were considered valid for implementation after this check.

## Results

During the discussion meetings undertaken together with planners and clinicians throughout the schedule construction process (Fig 1), practical infeasibilities of the schedule output by the MILP model were identified (and solved) in an iterative manner. In order to overcome those limitations, the MILP model structure and/or input parameters were modified accordingly until an implementable final schedule could be obtained. Table 3 shows the main infeasibilities and limitations found during the schedule construction process, as well as the solutions found to overcome them. We use the notation and constraints of the MILP model (S1 Appendix) to describe the solutions.

**Table 3:** Main infeasibilities and limitations found during the schedule construction method and corresponding model adjustments.

Unfeasibility/limitation	Solution found to overcome limitation	RT center(s)
Linacs’ maintenance times	Set linac as unavailable during maintenance hours using parameters *a*_*kst*_	NKI, BVI
Treatment sessions of some patients must fall within a restricted time window (e.g. 08h30-17h00) in order to guarantee that specialized staff are present during the sessions of the applicable patients	Add constraints (16)	NKI, BVI
Patients with 2 sessions per day (6h of interval is necessary between same-day sessions)	Create one dummy (fictitious) patient and insert time window constraints between the daily sessions of those two patients (using constraints (16))	BVI, NKI
Certain linac(s) cannot be assigned to certain patient(s) (e.g. due to lack of cone-beam CT technology)	Update $K^{i}$ accordingly	NKI
Staff breaks	Set linac as unavailable during staff breaks by setting parameters *a*_*kst*_ accordingly	BVI
Account for treatments that need to start on a Monday	Set due date *d*_*i*_ to Monday	NKI, BVI
Maximum number of patients starting treatment in the same linac, same day is limited to 6	Add constraints (5–7) with *C* = 6	NKI
5-min empty time slot at the end of every hour to accommodate possible delays	Set the last time slot of every hour as unavailable (using *a*_*kst*_) in each linac	BVI

Tables 4 and 5 show the performance of the final schedule obtained by the MILP model after it has been considered validated by each RT center, in comparison with the solution manually built and implemented in practice by the corresponding center on that same week. In either test case, all new patients started treatment within their target due date, both in practice and in the model’s solution. Moreover, the number of patients switching between different linacs throughout the course of their treatment decreased from 71 to 0 (NKI), and from 43 to 2 (BVI) with our model. In the NKI case, all performance indicators improved substantially, although a fewer number of sessions have been scheduled by the model (795) than was done in practice (807). Since average waiting times are not represented in the objective function but in the form of constraints in our model, more patients than in practice start treatment at (but not earlier than) the due date defined by clinicians for the particular patient’s trajectory. Moreover, as we can see in the bottom row of Table 4, there is enough capacity to accommodate fluctuations in the number of sessions scheduled per week, if needed, since linac utilization rates average 78–80%. Although we found that the solution found in practice provides slightly lower average waiting times for the start of treatment of new patients, we also verified that not only the variation is marginal (around 1%), but also that new patients are ensured a start of treatment that meets the recommended national guidelines in all cases.

**Table 4:** Performance comparison between the schedule built manually and the MILP model in the NKI.

	Practice	Model
No. sessions scheduled	807	795
No. new patients starting treatment within national targets	48 (100%)	48 (100%)
No. sessions within the time window requested by patient	n.a.	776 (97.6%)
Average standard deviation between sessions (min)	103.0	50.4
Median standard deviation between sessions (min)	91.9	43.2
No. patients switching linacs	71	0
Daily average linac utilization rate (SD)	0.80 (0.16)*	0.78 (0.10)*

*excludes linac L6 since it is a “back-up” linac only used for a small subset of patients (daily average utilization is approx. 0.15 in both cases).

**Table 5:** Performance comparison between the schedule constructed manually and the MILP model in the BVI.

	Practice	Model
No. sessions scheduled	232	232
No. new patients starting treatment within national targets	6 (100%)	6 (100%)
No. gaps in the schedule	18	5
Average standard deviation between sessions (min)	94.8	54.5
Median standard deviation between sessions (min)	86.4	60.7
No. patients switching linacs	43	2
Daily average linac utilization rate (SD)	0.65 (0.11)	0.67 (0.12)

Although data regarding the fulfillment of patient preferences have so far not been recorded in practice, the model was able to schedule 98% of the treatment sessions within the desired time window. Moreover, time variation amongst appointment times for the same patient decreased by 51% from an average SD of 103.0 to 50.4 minutes. The same tendency is observed in the median indicator, with the median patient seeing their standard deviation decrease from 91.9 to approximately 43.2 minutes with our model. A total of 1 hour and 27 minutes of computation time was needed to obtain the final schedule for the NKI case.

Results for the BVI case show that the number of sessions scheduled by the final MILP model matched the ones scheduled in practice at 232, resulting in a similar average utilization rate of linacs and an approximate daily standard variation. The solution obtained by using the model was able to provide a more compact schedule with as few as 5 gaps, as opposed to 18 gaps counted in the solution produced by the actual planners. The MILP model was also able to decrease the average standard deviation between appointments times by 43% from 94.8 to 54.5 and the median standard deviation reduce from 86.4 to 60.7 minutes, thus improving the consistency of appointment times. For the BVI case, the solution could be found in just 5 minutes of computation time. All instances have been solved to optimality.

## Discussion

The use of a mathematical OR model allowed to find a solution for the RT scheduling problem in both centers that proved both feasible and acceptable to the local staff. Several adjustments, performed in a stepwise manner, were necessary in order to improve and validate the MILP model before the output solution could be considered implementable. We could improve the weekly schedule for radiation sessions while optimizing the measured KPIs in both centers. In the NKI case, fewer sessions have been scheduled by the model than in practice (795 vs 807). Since maximizing the number of sessions delivered is not an objective in our model, the optimization process has no incentive to anticipate the start of treatment to an earlier date but will rather use the available capacity to maximize the satisfaction of patient preferences and ensure that all the medical and technological constraints are fulfilled, which has been verified. However, in practice, we observed that for a small number of new patients, treatment courses started earlier than the pre-defined due date, leading to a few more sessions scheduled than with our model. Small fluctuations in the number of sessions from week to week will not be an issue according to our results, since not only there is enough spare capacity to accommodate them, with average linac utilization rates varying between 65%-80%, but also because the obtained solutions result in a more balanced workload between linacs than in practice, at least for the NKI case (see Table 4). In case of RT centers interested in maximizing the number of sessions delivered, adjustments can be easily performed by either setting more ambitious due dates, or by adding a constraint that sets a lower bound on the number of sessions delivered on that specific week for the center a whole. In the former situation, a new patient having Wednesday as its target starting date, can in reality start treatment on Monday instead, leading to two extra sessions scheduled by the model. In the latter case, the lower bound would have to be assessed based on the total number of patients to be scheduled and the prescribed number of weekly sessions for each of those patients in order to obtain a range of total sessions that can be delivered on the planning week. The planner could then define the target value (lower bound) within that feasible range by trading-off timeliness and fulfilment of patient preferences.

Although actual patient preferences could not be obtained from the NKI clinic as they were not being recorded in practice, our model was able to fulfill the estimated preferences a total of 98% of the times. A sensitivity analysis on the impact of varying the preferential time window distributions may be useful to further assess the robustness of the model [21]. Moreover, the number of gaps in the schedule decreased by 72% in the BVI, with the daily number of treatment sessions being delivered earlier in the day during the whole week. This allows staff members to dedicate part of their time to other tasks. The consistency amongst the appointment times has also improved considerably with our MILP model, with the average standard deviation between appointment times decreasing by 51% and 43% for the NKI and BVI cases, respectively. This offers much more consistency for patients planning their daily calendar.

Our tool has proved effective in reducing the planning time needed to construct a schedule for RT treatment sessions. Every week, planners need to have finalized a general schedule of treatment sessions to be realized on the following week. In the NKI, for instance, 2–3 planners take about 1.5 days to create such a schedule. Instead, using our tool and up-to-date patient data, a planner can obtain the optimal solution that maximizes patient preferences for the whole patient population. Thereafter, the planner would only need to validate, and possibly manually adjust, the final solution before it can be communicated to patients. We estimate the complete process can be finalized in a time frame of 2–4 hours, and the final solutions would be considerably more efficient that the ones currently produced manually in RT clinics. In very large RT centers where problem size increases complexity to a significant degree, a heuristic procedure may be applied to pre-allocate patients to (subgroups of) linacs before the MILP model is applied to solve the problem for each subgroup of linacs independently [21]. By providing a decision-making tool that generates a schedule in an automated way, we not only save planners the burden of having to produce a feasible schedule in due time, but also allows them to prioritize their activities more easily. Nevertheless, a limitation in our computational experiments is that a one-week planning problem is solved, when RT treatments may take several weeks of daily sessions for some patients. In our model, a continuation of the previous week’s schedule for patients who already are under treatment can be achieved with little effort by setting the parameters of each patient regarding the time window for treatment sessions and the linac on which the patient is being treated. These parameters $t_{i}^{\min},t_{i}^{\max}$ and *c*_*i*_ are obtained from the schedule output by the model on the previous week. Another limitation of our results concerns the full validation of our output solutions. Checking all scheduled appointments for validation is a very cumbersome task that takes a considerable amount of the planners’ time. Moreover, the system does neither allow easy export nor import of the patient data in a way that it is accessible amongst the several involved personnel across the hospital, and the implementation and communication of the solution to patients have to be done in a manual way, requiring a considerable amount of time from the actual planners (over 50 hours only for the NKI case) which was, unfortunately, not available in our project. Nevertheless, we believe that the trial-and-error procedure performed for model adjustments, plus the validation performed in a significant portion of the patient population are enough to ensure the robustness of our tool.

Although the application of the mathematical programming model resulted in substantial performance improvements, the obtained schedule may still need to be further (manually) improved “*a posteriori*”. This may in fact be necessary to address specific needs of certain patients. Some patients may have individual characteristics that are not possible to be implemented in the model, and others are so specific that the completeness of the output solution would not tradeoff the additional computational complexity brought into the model if integrating them. For example, some patients do not speak either English or Dutch, which requires the presence of a translator in the room who has a limited contracted time availability. Some other patients may want to be treated before 9h00 every day except on Thursday where they prefer their timeslot after 16h00 for some reason. Since these patient-specific preferences and characteristics happen so rarely (less than 1% of the times), it is rather beneficial that they are manually introduced after a solution has been found by our model rather than incorporate them in the model. Although they did not prove critical for the RT centers at study, these and other features related to patient-centeredness, such as the possibility of choosing the staff members or the treatment starting date, can be further included into the model to maximize overall patient satisfaction for other RT centers. Other features may be added to the model, such as the coordination between the radiation sessions and other appointments that patients may need. For instance, patients are often scheduled for a weekly follow-up consultation with the doctor. Scheduling this consultation with a minimal time gap between the start/end of the radiation session results in a minimal duration the patient has to be at the hospital. Scheduling in such way would be of added value for patients and for the hospital. Gradually, the coordination between treatment sessions and other appointment types (e.g. chemotherapy appointments, blood analysis, a follow-up CT scan, mouth hygienist, etc.) can be added to the MILP model for the development of a more holistic planning tool. We estimate that these changes would require an additional planning time ranging between one and two hours when performing a manual rescheduling of some sessions.

While healthcare institutions have to strive for efficiency and stakeholders demand excellence in the delivery of care, we conclude that operations research tools can certainly be considered for implementation as they have demonstrated to be able to contribute to improved performance of treatment facilities. We achieved this by supporting planners with (theoretical) evidence-based tools and took them along in a stepwise and interactive implementation process, overcoming the use of traditional planning methods that provide sub-optimal solutions for both patients and resources [22]. By achieving a schedule that has been verified by the actual planners until it was considered ready for implementation, we take a significant step in the implementation of OR models in clinical practice compared to the current literature [13]. The next steps would be to have the OR model running on a weekly basis in each clinic for a certain period of time and perform a pre-post performance evaluation to assess the accuracy of our results. However, the manual export and import of data and output solutions to and from the patient scheduling systems, or the development of a user-friendly, bug-free computer application that can read and write the inputs and outputs of the OR model in an automated way are thorough processes that could not be performed within the research project timeline. Nevertheless, in our experience we found that a gradual development/adjustment of the OR models, in small steps, is recommended for a smooth translation of those models into the clinic. Moreover, the engagement of all stakeholders from the very beginning of the study has allowed to create a collaborative environment based on constant communication and mutual confidence that were crucial for the realization of the implementation steps. Bringing the OR specialists, planners, managers and clinicians together as part of a project team with regular meetings, where model adaptations and new results have been made easy to visualize and interpret, has helped bridge the gap between the different professionals involved.

Since the RT scheduling processes designed for this study are rather standard, we believe that our intervention would produce similar results in other RT centers. Moreover, the MILP model can be adjusted by adding/removing constraints on a necessity basis according to the characteristics and workflow of the RT center aiming to implement it. To this end, the process of adjustment and validation of OR models proposed in this work can be used by healthcare institutions to promote implementation efforts of OR-based methodologies into clinical practice.

## Conclusions

The application of an OR model for RT treatment scheduling has proven to be capable of supporting RT planners produce a high-quality schedule that satisfies all medical and technical constraints in a much faster way than currently done in practice. In our study, the early engagement of managers and clinicians, staying in close contact with stakeholders, and performing stepwise model adaptions in small steps proved to be the most important factors to facilitate the implementation of an OR model in the practice setting.

## Supporting information

S1 Appendix MILP model used to generate a schedule for the RT treatment scheduling problem. (DOCX)

S1 Data (XLSX)

## References

[1] Bray F, Ferlay J, Soerjomataram I, Siegel RL, Torre LA, Jemal A. Global cancer statistics 2018: GLOBOCAN estimates of incidence and mortality worldwide for 36 cancers in 185 countries. CA: A Cancer Journal for Clinicians. 2018;68(6):394–424. 30207593 10.3322/caac.21492

[2] Borras JM, Lievens Y, Barton M, Corral J, Ferlay J, Bray F, et al How many new cancer patients in Europe will require radiotherapy by 2025? An ESTRO-HERO analysis. Radiotherapy and Oncology. 2016;119(1):5–11. 10.1016/j.radonc.2016.02.016 26922487

[3] Vitoux A, Grenier C, Lartigau E. 147 Improvement in the quality of practices in radiotherapy: the regular measurement of indicators. 2010;19(Suppl 1):A170–A1.

[4] Chen Z, King W, Pearcey R, Kerba M, Mackillop WJ. The relationship between waiting time for radiotherapy and clinical outcomes: A systematic review of the literature. Radiotherapy and Oncology. 2008;87(1):3–16. 10.1016/j.radonc.2007.11.016 18160158

[5] Mackillop WJ. Killing time: The consequences of delays in radiotherapy. Radiotherapy and Oncology. 2007;84(1):1–4. 10.1016/j.radonc.2007.05.006 17574695

[6] Olivotto IA, Soo J, Olson RA, Rowe L, French J, Jensen B, et al Patient preferences for timing and access to radiation therapy. Curr Oncol. 2015;22(4):279–86. 10.3747/co.22.2532 26300666 PMC4530813

[7] Petersen GS, Knudsen JL, Vinter MM. Cancer patients’ preferences of care within hospitals: a systematic literature review. International Journal for Quality in Health Care. 2015;27(5):384–95. 10.1093/intqhc/mzv059 26265160

[8] Petrovic D, Castro E, Petrovic S, Kapamara T. Radiotherapy Scheduling In: Uyar AS, Ozcan E, Urquhart N, editors. Automated Scheduling and Planning: From Theory to Practice. Berlin, Heidelberg: Springer Berlin Heidelberg; 2013 p. 155–89.

[9] Vieira B, Demirtas D, B. van de Kamer J, Hans EW, van Harten W. Improving workflow control in radiotherapy using discrete-event simulation. BMC Medical Informatics and Decision Making. 2019;19(1):199 10.1186/s12911-019-0910-0 31651304 PMC6814107

[10] Vieira B, Demirtas D, van de Kamer JB, Hans EW, van Harten W. A mathematical programming model for optimizing the staff allocation in radiotherapy under uncertain demand. European Journal of Operational Research. 2018;270(2):709–22.

[11] Saville CE, Smith HK, Bijak K. Operational research techniques applied throughout cancer care services: a review. Health Systems. 2019;8(1):52–73. 10.1080/20476965.2017.1414741 31214354 PMC6507866

[12] Abdur R, Ana V. Operations Research in Healthcare: a survey. International Transactions in Operational Research. 2011;18(1):1–31.

[13] van Lent WA, Sanders EM, van Harten WH. Exploring improvements in patient logistics in Dutch hospitals with a survey. BMC health services research. 2012;12:232 10.1186/1472-6963-12-232 22852880 PMC3496592

[14] Brailsford SC, Harper PR, Patel B. An analysis of the academic literature on simulation and modelling in health care. J Simul. 2009;3:130–40.

[15] Vieira B, Hans EW, van Vliet-Vroegindeweij C, van de Kamer J, van Harten W. Operations research for resource planning and -use in radiotherapy: a literature review. BMC Med Inform Decis Mak. 2016;16(1):149 10.1186/s12911-016-0390-4 27884182 PMC5123361

[16] Bradley BD, Jung T, Tandon-Verma A, Khoury B, Chan TCY, Cheng Y-L. Operations research in global health: a scoping review with a focus on the themes of health equity and impact. Health Research Policy and Systems. 2017;15(1):32 10.1186/s12961-017-0187-7 28420381 PMC5395767

[17] Vieira B, Demirtas D, van de Kamer JB, Hans EW, Rousseau L-M, Lahrichi N, et al Radiotherapy treatment scheduling considering time window preferences. Health Care Management Science. 2020 10.1007/s10729-020-09510-8 32594285 PMC7676074

[18] ELEKTA. 2020 [Available from: https://www.elekta.com/software-solutions/care-management/mosaiq-radiation-oncology/.

[19] VARIAN. 2020 [Available from: https://www.varian.com/products/software/information-systems/aria-ois-medical-oncology.

[20] NVRO. Waiting times, standards and maximum waiting times for radiotherapy (in dutch) 2000 [Available from: http://www.nvro.nl/kwaliteit/indicatoren.

[21] Vieira B, Demirtas D, van de Kamer JB, Rousseau L-M, Lahrichi N, Hans EW, et al Radiotherapy treatment scheduling considering time window preferences. Health Care Management Science. 2020 10.1007/s10729-020-09510-8 32594285 PMC7676074

[22] van Bodegom-Vos L, Davidoff F, Marang-van de Mheen PJ. Implementation and de-implementation: two sides of the same coin? BMJ Quality & Safety. 2017;26 (6):495–501. 10.1136/bmjqs-2016-005473 27512102

