# Supplementary material for: Radiotherapy treatment scheduling: Implementing operations research into clinical practice
Source: PLoS One. 2021 Feb 19;16(2):e0247428. doi: 10.1371/journal.pone.0247428 (PMC7894882; doi:10.1371/journal.pone.0247428)
Supplement: S1 Appendix — (DOCX) [file pone.0247428.s001.docx]

# S1 Appendix– MILP model used to generate a schedule for the RT treatment scheduling problem

In this appendix, we present the mixed-integer linear programming (MILP) model used to find a feasible and optimized schedule for the RT scheduling problem. The capacity of each linac is divided in time slots $s=1,\ldots,\left| \mathcal{S} \right|$ of a pre-defined, fixed duration $l$. Patients' sessions, when scheduled, are assigned to a certain starting time slot on a certain linac and day. If a certain starting slot is assigned to a patient, we prevent the next slots needed to achieve the corresponding patient's session duration on that same linac and day from being assigned to other patients.

***Input parameters***

We use the following notation and input parameters to formulate the problem:

| $\mathcal{P}$ | set of patients to be scheduled ($i,j\in\mathcal{P}$) |
| --- | --- |
| $\mathcal{K}$ | set of linear accelerators ($k\in\mathcal{K}$) |
| $\mathcal{S}$ | set of time slots available per linac, per time period ($s\in\mathcal{S}$) |
| $\mathcal{T}$ | set of time periods (days) in the planning horizon ($t\in\mathcal{T}$) |
| $\mathcal{P}^{\mathcal{n}}$ | set of patients who have not started treatment ($\mathcal{P}^{\mathcal{n}}\subseteq\mathcal{P}$) |
| $\mathcal{P}^{\mathcal{m}}$ | set of patients who must start treatment on Monday ($\mathcal{P}^{\mathcal{m}}\subseteq\mathcal{P}$) |
| $\mathcal{P}^{\mathcal{f}}$ | set of patients with restricted time frame for treatment sessions ($\mathcal{P}^{\mathcal{f}}\subseteq\mathcal{P}$) |
| $\mathcal{K}^{\mathcal{i}}$ | set of feasible linacs for treating patient $i$ ($\mathcal{K}^{\mathcal{i}}\subseteq\mathcal{K}$) |
| $C$ | maximum number of patients starting treatment in the same linac and same day |
| $l$ | time slot duration, in minutes, in each linac, each day |
| $a_{kst}$ | 1 if slot $s$of linac $k$ is available on time period$t,$0 otherwise |
| $\underline{f}^{t},\overline{f}^{t}$ | lower and upper bound of the restricted time frame set for time period $t$ |
| $I_{i}$ | number of total remaining sessions to be delivered to patient $i$ |
| $d_{i}$ | due date: time period by which patient $i$ must start treatment |
| $p_{i}$ | duration, in number of time slots, of each session of patient $i$ |
| $b_{i}$ | number of time periods needed between sessions of patient $i$ (1 for consecutive daily sessions) |
| $t_{i}^{\text{min}},t_{i}^{\text{max}}$ | lower and upper bound of the time window preference for patient $i$ |
| $c_{i}$ | linac in which patient $i\notin\mathcal{P}^{\mathcal{n}}$ is currently undergoing treatment |

***Decision variables***

We use a set of binary variables $x_{\mathrm{iks}}^{t},$which take the value 1 if patient $i$ is scheduled for a session starting on time slot $s$ of linac $k$, in day $t$, and 0 otherwise. Real variables $\Delta_{\mathrm{it}}^{-}$and $\Delta_{\mathrm{it}}^{+}$ are used to represent the deviations from the intended sessions’ starting time for each patient, in each day. Binary variables $y_{\mathrm{ik}}^{t}$ are auxiliary variables, which will be equal to 1 if a new patient starts his/her treatment in period $t$ and linac $k$, and 0 otherwise.

| $x_{iks}^{t}$ | 1 if patient $i$ is scheduled a session starting on time slot $s$ of linac $k$ in day $t$, 0 otherwise |
| --- | --- |
| $y_{ik}^{t}$ | 1 if new patient $i$ starts treatment in period $t$ and linac $k$, 0 otherwise |
| $\Delta_{it}^{-},\Delta_{it}^{+}$ | lower and upper deviation, in minutes, from preference time window of patient $i$ in time period $t$ |

***Objective function(s)***

The objective function to be used in the MILP model will depend on the goal(s) of each RT center:

| Minimize the overall deviation between the bounds of the preferred time window $\left[ t_{i}^{\text{min}},t_{i}^{\text{max}} \right]$ given by patients and the starting time of their appointments:  $\min\sum_{i\in\mathcal{P}} \sum_{t\in\mathcal{T}} \left( \Delta_{it}^{-}+\Delta_{it}^{+} \right)$ | (1a) |
| --- | --- |
| Minimize the overall starting time of radiation sessions (thus scheduling them together and as early as possible):  $\min\sum_{i\in\mathcal{P}} \sum_{k\in\mathcal{K}} \sum_{s\in\mathcal{S}} \sum_{t\in\mathcal{T}} s.x_{iks}^{t}$ | (1b) |

***Constraints***

The values of the decision variables are bounded by a set of linear inequalities, which represent the practical constraints of the problem, as follows:

| Limit the number of sessions that each patient can receive to a maximum of one per day:  $\sum_{k\in\mathcal{K}} \sum_{s\in\mathcal{S}} x_{iks}^{t}\leq1,\forall i\in\mathcal{P,}\forall t\in\mathcal{T}$ | (2) |
| --- | --- |
| Ensure that each (available) slot of each linac is scheduled at most one session per day:  $\sum_{i\in\mathcal{P}} x_{iks}^{t}\leq a_{kst},\forall k\in\mathcal{K,}\forall s\in\mathcal{S,}\forall t\in\mathcal{T}$ | (3) |
| Each patient is assigned to a feasible linac, by preventing sessions of being assigned to slots of linacs that do not belong to $\mathcal{K}^{\mathcal{i}}$:  $\sum_{s\in\mathcal{S}} \sum_{t\in\mathcal{T}} x_{iks}^{t}\leq0,\forall i\in\mathcal{P,}\forall k\in\mathcal{K}\setminus\{\mathcal{K}^{\mathcal{i}}\}$ | (4) |
| Force $y_{ik}^{t}$variables to take the value 1 if a new patient $i$starts treatment on linac $k$ and day $t$:  $y_{ik}^{t}\geq\sum_{s\in\mathcal{S}} x_{iks}^{t}-\sum_{s\in\mathcal{S}} x_{iks}^{t^{'}},\forall i\in\mathcal{P}^{\mathcal{n}},\forall k\in\mathcal{K,}\forall t=2,..,\mathcal{T,}t^{'}=\max\{1,t-b_{i}\}$  $y_{ik}^{1}\geq\sum_{s\in\mathcal{S}} x_{iks}^{1},\forall i\in\mathcal{P}^{\mathcal{n}},\forall k\in\mathcal{K}$ | (5)  (6) |
| Limit the number of patients starting treatment in the same linac and same day to the pre-defined threshold $C:$  $\sum_{i\in\mathcal{P}^{\mathcal{n}}} y_{ik}^{t}\leq C,\forall k\in\mathcal{K,}\forall t\in\mathcal{T}$ | (7) |
| Restrict the number of sessions delivered during the planning horizon to the number of remaining sessions for that patient:  $\sum_{k\in\mathcal{K}} \sum_{s\in\mathcal{S}} \sum_{t\in\mathcal{T}} x_{iks}^{t}\leq I_{i},\forall i\in\mathcal{P}$ | (8) |
| Ensure that patients receive their sessions on the same linac and with the required frequency $b_{i}$ until the number of sessions or the end of planning horizon is reached:  $\sum_{s\in\mathcal{S}} x_{iks}^{t}-\sum_{s\in\mathcal{S}} \sum_{t^{'}=1}^{t-1} x_{iks}^{t^{'}}\leq\sum_{s\in\mathcal{S}} x_{iks}^{n},\forall i\in\mathcal{P,}\forall k\in\mathcal{K,}\forall t=2,..,\mathcal{T,}\forall n=t+b_{i},t+2b_{i},\ldots,\min\{\left\vert\mathcal{T} \right\vert,t+b_{i}\left( I_{i}-1 \right)\}$  $\sum_{s\in\mathcal{S}} x_{iks}^{1}\leq\sum_{s\in\mathcal{S}} x_{iks}^{n},\forall i\in\mathcal{P,}\forall k\in\mathcal{K,}\forall n=b_{i}+1,2b_{i}+1,\ldots,\min\{\left\vert\mathcal{T} \right\vert,b_{i}\left( I_{i}-1 \right)+1\}$ | (9)  (10) |
| Force the all the necessary sessions to be booked, at least every $b_{i}$ days, as soon as a first session is scheduled:  $1-\sum_{s\in\mathcal{S}} x_{iks}^{t}\geq\sum_{s\in\mathcal{S}} x_{iks}^{n},\forall i\in\mathcal{P,}\forall k\in\mathcal{K,}\forall t=1,..,\left\vert\mathcal{T} \right\vert-b_{i},\forall n=t+1,\ldots,t+b_{i}-1,b_{i}\geq2$ | (11) |
| Avoid unnecessary sessions from being scheduled in days occurring between the days of the sessions booked by constraints (9)-(10) when $b_{i}$ > 1:  $1-\sum_{s\in\mathcal{S}} x_{iks}^{t}\geq\sum_{s\in\mathcal{S}} x_{iks}^{n},\forall i\in\mathcal{P,}\forall k\in\mathcal{K,}\forall t=1,..,\left\vert\mathcal{T} \right\vert-b_{i},\forall n=t+1,\ldots,t+b_{i}-1,b_{i}\geq2$ | (12) |
| Impose that every patient starts treatment before their due date $d_{i}$ (note that for patients starting on Monday we can set $d_{i} = 1$):  $\sum_{k\in\mathcal{K}} \sum_{s\in S} \sum_{t=1}^{d_{i}} x_{iks}^{t}\geq1,\forall i\in\mathcal{P}$ | (13) |
| Prevent the remainder slots needed to achieve the session duration $p_{i}$ after the chosen starting slot $x_{iks}^{t}$ from being assigned to other patients on the same linac and day:  $x_{iks}^{t}\leq1-\sum_{i^{'}\in\mathcal{P}} x_{i^{'},k,s^{'}}^{t},\forall i\in\mathcal{P,}\forall k\in\mathcal{K,}\forall s=1,\ldots,\left\vert\mathcal{S} \right\vert-p_{i}+1,\forall t\in\mathcal{T,}\forall s^{'}=s+1,\ldots,s+p_{i}-1,p_{i}\geq2$ | (14) |
| Ensure that the starting slot of sessions with a duration of two or more slots are not assign to the last slot(s) of the day:  $x_{iks}^{t}=0,\forall i\in\mathcal{P,}\forall k\in\mathcal{K,}\forall s=\left\vert\mathcal{S} \right\vert-p_{i}+2,\ldots,S,\forall t\in\mathcal{T,}p_{i}\geq2$ | (15) |
| Ensure that sessions of each patient fall within the restricted time frame set by the department due to the need of ensuring that specialized staff are present during the sessions of the applicable patients ($\mathcal{P}^{\mathcal{f}}$):  $x_{iks}^{t}\leq0,\forall i\in\mathcal{P}^{\mathcal{f}},\forall k\in\mathcal{K,}\forall s\in\mathcal{S,}\forall t\in\mathcal{T,}s<\underline{f}^{t},s>\overline{f}^{t}$ | (16) |
| Force variables $\Delta_{it}^{-}$ and $\Delta_{it}^{+}$to take a non-zero value if a session's starting time deviates from the desired lower and upper bounds, respectively:  $t_{i}^{\text{min}}x_{iks}^{t}-\Delta_{it}^{-}\leq l\left( s-1 \right)x_{iks}^{t}\leq t_{i}^{\text{max}}x_{iks}^{t}+\Delta_{it}^{+},\forall i\in\mathcal{P,}\forall k\in\mathcal{K,}\forall s\in\mathcal{S,}\forall t\in\mathcal{T}$ | (17) |
| Non-negativity constraints associated with the real variables:  $\Delta_{it}^{-}\geq0,\Delta_{it}^{+}\geq0,\forall i\in\mathcal{P,}\forall t\in\mathcal{T}$ | (18) |
| Binary variables can only take the value 0 or 1:  $x_{iks}^{t},y_{ik}^{t}\in\{0,1\},\forall i\in\mathcal{P,}\forall k\in\mathcal{K,}\forall s\in\mathcal{S,}\forall t\in\mathcal{T}$ | (19) |

The MILP model was coded in C++ using Visual Studio 2017 and the Concert Technology of CPLEX v12.8.0, which was used as a solver. All experiments were conducted on a desktop computer with a processor Intel i7 3.6 GHz and 16 GB of RAM using up to 8 threads, running on a 64-bit version of Windows 10. A time limit of 28800 seconds (8 hours) of CPU was set for each computational experiment.
